# Comparison of once-daily versus twice-daily combination of Ropinirole prolonged release in Parkinson’s disease

**DOI:** 10.1186/1471-2377-13-113

**Published:** 2013-09-02

**Authors:** Ji Young Yun, Han-Joon Kim, Jee-Young Lee, Young Eun Kim, Ji Seon Kim, Jong-Min Kim, Beom S Jeon

**Affiliations:** 1 Department of Neurology, Ewha Womans University Mokdong Hospital, Seoul, Korea; 2 Department of Neurology, College of Medicine, Seoul National University, Seoul, Korea; 3 Movement Disorder Center, Seoul National University Hospital, Seoul, Korea; 4 Department of Neurology, Seoul National University-Seoul Metropolitan Government Boramae Medical Center, Seoul, Korea; 5 Department of Neurology, Seoul National University Bundang Hospital, Sungnam, Korea; 6 Department of Neurology, Chungbuk National University Hospital, Cheongju, Korea

**Keywords:** Parkinson’s disease, Motor control, Movement disorders, Dopamine agonist

## Abstract

**Background:**

Ropinirole prolonged release (RPR) is a once-daily formulation. However, there may be individual pharmacokinetic differences so that multiple dosing may be preferred in some individuals. This study compares once-daily and twice-daily RPR in patients with Parkinson’s disease.

**Methods:**

This study was an open-label crossover study. We enrolled Parkinson’s disease patients on dopamine agonist therapy with unsatisfactory control such as motor fluctuation, dyskinesia and sleep-related problems. Agonists were switched into equivalent dose of RPR. Subjects were consecutively enrolled into either once-daily first or twice-daily first groups, and received the same amount of RPR in a single and two divided dosing for 8 weeks respectively in a crossover manner without a washout period.

The primary outcome was a questionnaire of the preference completed by patients in the last visit. The secondary outcome measures included the Unified Parkinson’s Disease Rating Scale part 3 (mUPDRS), Hoehn and Yahr stage (H&Y); sleep questionnaire including overall quality of sleep, nocturnal off symptoms and early morning symptoms; Epworth Sleep Scale (ESS); compliances and patient global impression (PGI).

**Results:**

A total of 82 patients were enrolled and 61 completed the study. 31 patients preferred twice-daily regimen, 17 preferred the once-daily regimen, and 13 had no preference. Their mean mUPDRS, H&Y, ESS, sleep quality, compliance and adverse events were not statistically different in both regimens. PGI-improvement on wearing off defined was better in twice-daily dosing regimen.

**Conclusions:**

RPR is a once-daily formulation, but multiple dosing was preferred in many patients. Multiple dosing of RPR might be a therapeutic option if once-daily dosing is unsatisfactory.

**Trial registration:**

This study is registered with ClinicalTrials.gov, number NCT00986245.

## Background

Ropinirole is a non-ergot D2/D3 agonist for the management of Parkinson’s disease (PD). Ropinirole, when given in the immediate-release (IR) form, has to be taken three times a day. It is hypothesized that pulsatile stimulation of dopamine receptors in PD may induce motor fluctuation [1]. Theoretically, motor fluctuation can be avoided by continuous stimulation of the dopamine receptors. Based on this hypothesis, a prolonged-release (PR) formulation was developed.

Ropinirole PR (RPR) is a once daily formulation of ropinirole that is not inferior to the immediate-release formulation [2]. In advanced PD patients, once-daily RPR shows significantly greater improvement in parkinsonian symptoms than IR from [3]. Once daily dosing provides better medication compliance [4] and smoother plasma levels than ropinirole IR (RIR) [5]. Therefore, this formulation is regarded as valuable addition to available antiparkinsonian medications [6,7].

However, we have met patients unsatisfied with once daily RPR who had asked for multiple daily dosing in clinics occasionally. As seen in the time window of the ropinirole level from Tompson et al. [5], the nocturnal concentration may be lower in the once-daily RPR than the three times daily RIR. The “off” symptoms between dusk and dawn might be more severe in the once daily RPR dosing than in the multiple daily RIR dosing. In addition, the early morning off duration may be longer in the RPR than in the RIR, since the increasing slope of the plasma concentration is gentler in the RPR. Thus multiple dosing with RPR may provide even better control in some patients.

Herein, we compared the preference of patients for once-daily versus twice-daily combination of RPR.

## Methods

### Patients

PD patients between 30 and 80 years of age were eligible for the study. All subjects were taking a stable dose of levodopa and dopamine agonist for at least 4 weeks prior to the screening. They were on dopamine agonist (RIR or pramipexole IR) and were considering changing to RPR due to suboptimal control with levodopa and dopamine agonist therapy, for example, motor fluctuation, dyskinesia, and sleep-related problems.

There were no limitations on other antiparkinsonian medications as long as the treatments remained stable for at least 4 weeks prior to and throughout the study. Neuroleptics were not allowed in this study. Patients with significant or uncontrolled psychiatric, cognitive, neurologic or other medical disorders; a history of severe dizziness or fainting due to orthostatic hypotension; a recent history or current evidence of drug abuse or alcoholism; a history of severe adverse events related dopaminergic agents; a history of allergic reaction to the similar medications; and a history of heavy metal poisoning were not eligible for the study. Patients were excluded from the study if they had used RPR, or an investigational medication within 4 weeks. We did not enroll patients who were on less than 2 mg of RIR or less than 0.375 mg of pramipexole IR since we could not prescribe a twice-daily dose without splitting the tablet shown in Additional file 1.

### Study design

This study was a two-centre, 16 week, two-period, open-label crossover study between once daily and twice daily dosing of RPR. The study protocol was approved by Institutional Review Board of the Seoul National University Hospital and the Seoul National University Metropolitan Boramae Hospital and conformed to the principles of the Declaration of Helsinki. All patients signed an informed consent before participation in this study.

**Figure 1:**
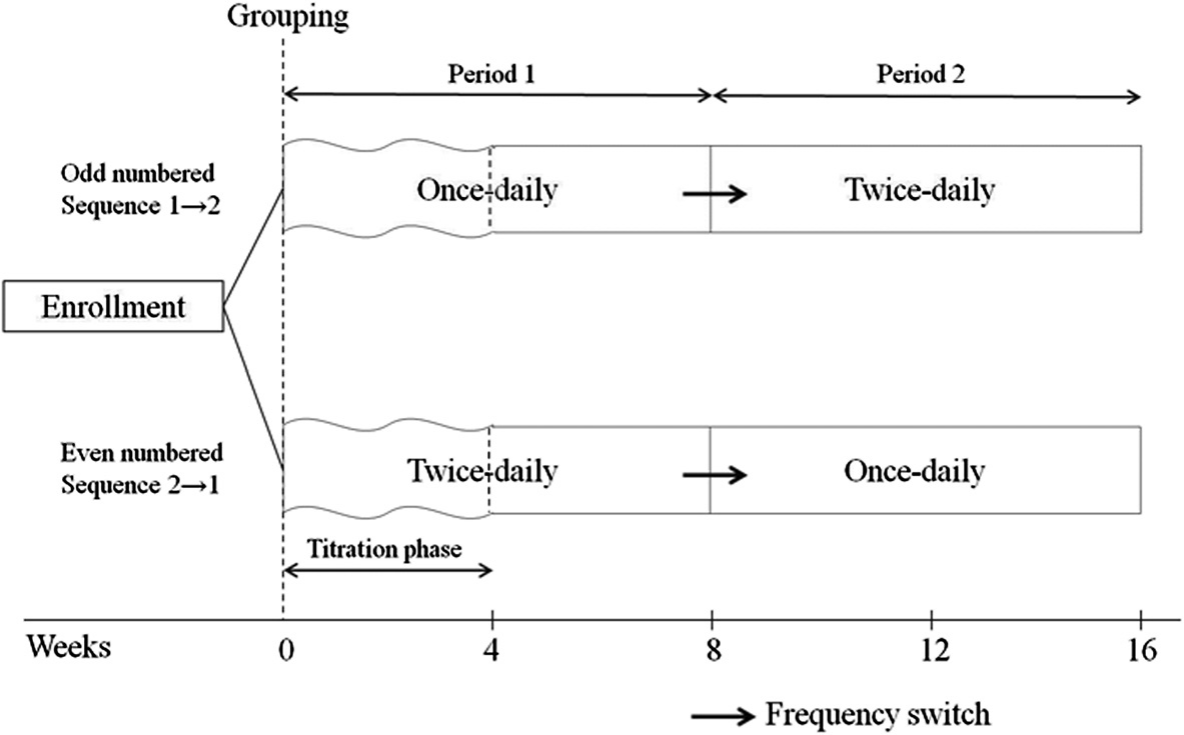
Study design.

Subjects were sequentially enrolled into once-daily dosing first or twice-daily dosing first group. Each group received 8-weeks of RPR once daily or twice daily and then without a washout period switched into twice daily or once daily dosing schedules for 8-weeks in an open label fashion (Figure 1).

Conversion ratio between RIR and RPR was 1:1 and pramipexole IR to RPR was 1:5. RPR comes in even numbered sizes (2, 4, 8 mg/tablets). Therefore, upward adjustment of the RPR dose was made when needed in order not to break the RPR tablets. For example, when RIR was given at 9 mg/d, RPR was given at 10 mg/d. In twice daily dosing, we split the dose of the RPR into two doses and split into unequal doses when needed in order not to break the RPR tablets (for example, 10 mg into 6 and 4 mg, Table 1). First dosing of RPR was given with the first dosing of the other antiparkinsonian medications. The timing and dose of the second dosing was based on clinical decision, which was usually in the evening or in the late afternoon at a lower dosage.

**Table 1:** Baseline characteristics

	**QD → BID (N = 29)**	**BID → QD (N = 32)**	***P *Value**	**Overall (N = 61)**
Age (years)	60.9 ± 9.8	61.1 ± 8.4	0.925	61.0 ± 9.0
Onset age	51.7 ± 10.8	51.8 ± 7.0	0.857	51.7 ± 8.9
Sex (M:F)	11:18	13:19	1.000	24:37
mUPDRS	20.0 ± 9.5	22.5 ± 8.6	0.170	21.3 ± 9.0
Hoehn and Yahr stage	2.1 ± 0.5	2.4 ± 0.7	0.063	2.2 ± 0.6
Ropinirole PR dose after titration	8.9 ± 5.6	11.0 ± 5.7	0.128	10.0 ± 5.7
LEDD	894.0 ± 392.8	865.9 ± 361.7	0.879	879.2 ± 373.9
Epworth Sleep Scale	6.1 ± 5.5	6.2 ± 5.9	0.353	6.1 ± 5.6
Sleep questionnaire				
Overall sleep quality	3.8 ± 2.6	2.9 ± 2.7	0.232	3.4 ± 2.7
Nocturnal off-symptoms	2.5 ± 2.8	3.4 ± 3.7	0.659	3.0 ± 3.3
Early morning off symptoms	2.9 ± 2.7	3.0 ± 3.9	0.721	3.0 ± 3.8

QD, Once-daily; BID, Twice-daily; mUPDRS, Unified Parkinson’s Disease Rating Scale part 3; PR, prolonged-release; LEDD, levodopa equivalent dose.

Titration of RPR was allowed only in the initial 4 weeks in the first period of each sequence. The dose was titrated until an optimal therapeutic response was achieved or intolerable adverse effects disappeared. The dosing frequency was maintained during the titration phase. Once an optimal dose was achieved, the subject was maintained on that dose for the remainder of the treatment phase. Changes in other antiparkinsonian medications were not allowed. In the second period in each sequence, the titration of RPR was not allowed. However, if the subject complained of intolerable off-symptoms, dyskinesia or adverse effects, early completion was accepted after the last visit earlier than the planned 16 weeks.

Scheduled study visits were baseline and week 8 and 16. Patients were allowed to make non-scheduled visits when needed. At the baseline visit, all subjects were assessed with the Unified Parkinson’s Disease Rating Scale part 3 (mUPDRS) and Hoehn and Yahr stage (H&Y) with a medication-on state, sleep questionnaire including overall quality of sleep, nocturnal off symptoms and early morning motor symptom; Epworth Sleep Scale (ESS). In the sleep questionnaire, we used the visual analogue scale ranging from 0 (perfect imaginable quality of sleep) to 10 (worst imaginable quality of sleep).

After 8 and 16 weeks or at last visit for early completion, all assessments were repeated and the Patient’s Global Impressions (PGI) and compliance to the prescribed doses were obtained. Medication compliance was recorded. During each period, mean compliance rates were calculated based on the total prescribed doses and total actually taken doses for all subjects during each period. Adverse events (AEs) and changes in wearing-off and dyskinesias were followed throughout the study. Preference for dosing schedule was asked at the completion of the study. The preference was assessed with a question: “which regimen do you prefer?” Available answers were “I prefer the once a day”; “I prefer the twice a day”; “I do not prefer one treatment over the other.” In addition, the reasons for the preference were also asked.

If subjects completed the study earlier than the completion of the second treatment period, they were asked for their preferences and evaluated at the discontinuation visit. Patients who discontinued during the first treatment period or did not complete the questionnaire for the preference were excluded from the analysis.

### Outcome measures

Primary outcome measure was the preference of the subjects between once-daily versus twice-daily of RPR at the completion or at early completion after crossover.

Secondary outcome measures included the mUPDRS and H&Y at the medication-on state; sleep questionnaire including overall quality of sleep, nocturnal off symptoms and early morning motor symptoms; ESS; Compliance; and PGI of improvement (PGI-I). Additional secondary outcome measures were the proportion of patients who answered ‘improvement’, ‘stationary’ or ‘aggravated’ for each period and the severity of the wearing-off and dyskinesia. As safety measures, AEs were followed throughout the study. This study is registered with ClinicalTrials.gov, number NCT00986245.

### Statistical analyses

Comparisons between the Sequences once daily to twice daily (1 → 2) and twice daily to once daily (2 → 1) for baseline were analyzed using Mann–Whitney test. For the primary analysis, descriptive statistics were used to determine subject preferences. Comparisons between groups that preferred once-daily or twice-daily were analyzed with Mann–Whitney tests. Comparisons between once-daily and twice-daily phases in mUPDRS, H&Y, PGI-I, sleep questionnaire, ESS, compliance and changes in the wearing-off and dyskinesias were analyzed with Wilcoxon signed rank comparisons. McNemar tests were used to evaluate differences in AE between once-daily and twice-daily phases.

Statistical analyses were done with SPSS statistical package version 19 (SPSS Inc., Chicago, IL, USA).

## Results

### Subjects and discontinuations

A total of 82 patients with PD were enrolled in this study at two centres in Seoul, Korea. The first subject was enrolled in September 2009 and the last subject completed the study in December 2010. Forty one patients started with once-daily dosing and another 41 patients with twice-daily dosing. Baseline demographics were much the same in both treatment groups (Table 1).

**Figure 2:**
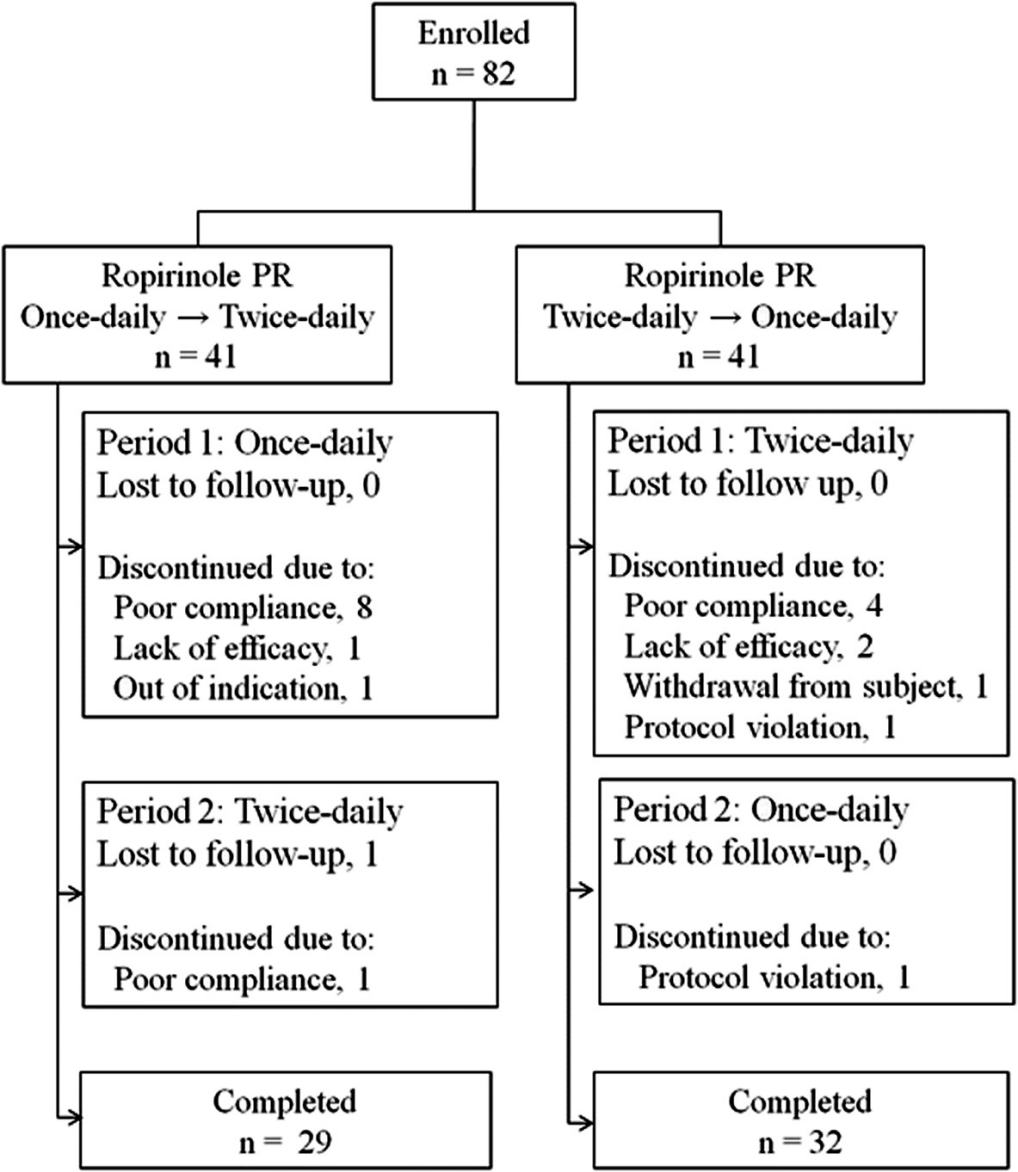
Subject flow chart.

Twenty one subjects (25.6%) did not complete this study (Figure 2). The most common reason for discontinuation was poor compliance. The discontinuation rate was higher in Sequence 1 → 2 (respectively, 12 in sequence 1 → 2 vs. 9 in sequence 2 → 1); however, it was not statistical significant. One patient was excluded because he was unknowingly on levosulpride. According to dosing frequency, ten subjects dropped out from the once-daily period and ten from the twice-daily period. According to the sequence, eighteen patients dropped out during the first period and three during the second period in each sequence. A total of sixty one subjects (74.4%) were included in the final analysis (Figure 2).

At the baseline visit, twenty four patients were on RIR (4.9 ± 2.2 mg/day) and thirty seven on pramipexole IR (2.4 ± 1.0 mg/day). Based on the conversion ratio, the dose of RIR and pramipexole IR was calculated as RPR 9.1 ± 5.0 mg/day. In order to avoid breaking the RPR tablets, the actual converted dose using Table 1 was 10.0 ± 5.7 mg/day before titration. After the titration, the average dose of RPR was 10.7 ± 6.2 mg/day.

### Primary outcome

**Figure 3:**
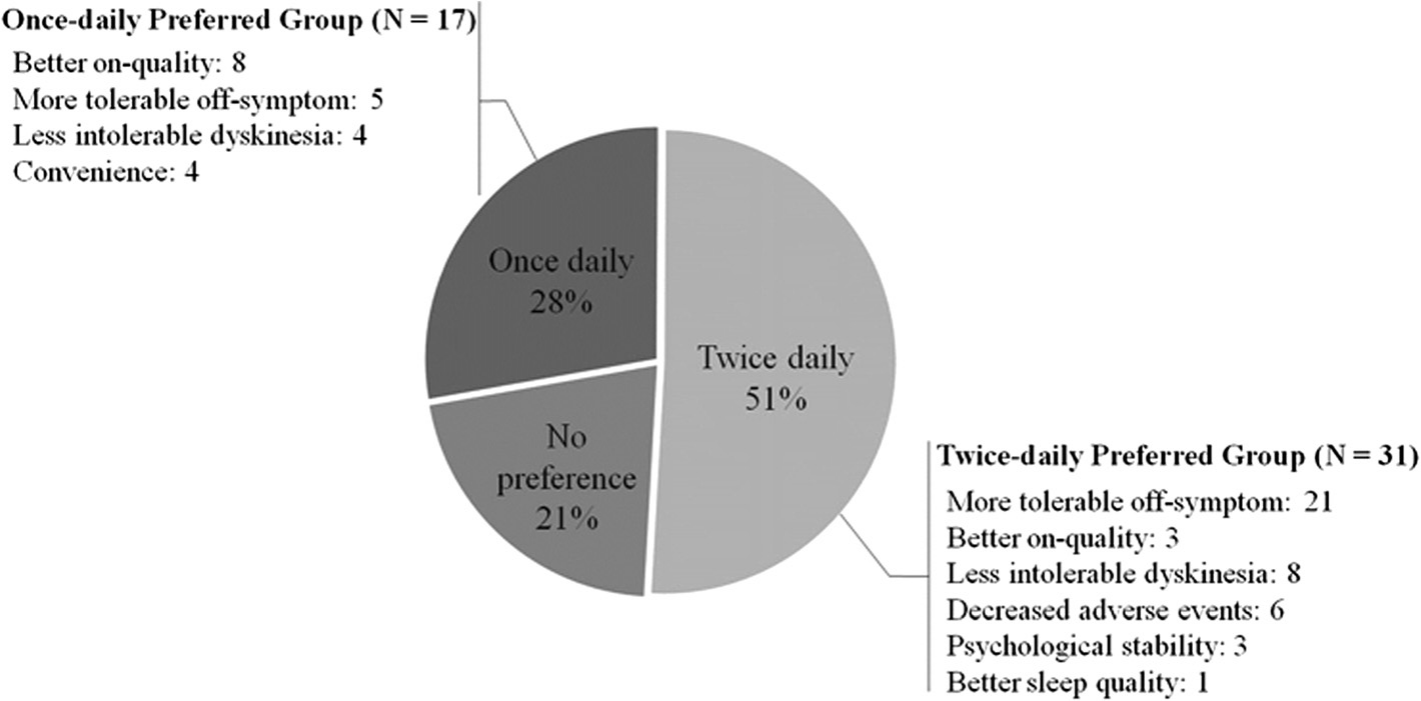
Patient’s preferences and reasons.

In response to the final preference questionnaire, 28% (n = 17) of the patients preferred the once-daily regimen, 51% (n = 31) preferred the twice-daily regimen, and 21% (n = 21) did not have a preference (Figure 3).

In the group that preferred the once-daily regimen (Figure 3), the preference reasons were better on-quality (n = 8), more tolerable off-symptoms (n = 5), less intolerable dyskinesia (n = 4) and convenience (n = 4). The reasons for preferring the twice-daily regimen were (Figure 3): more tolerable off-symptoms (n = 21), less intolerable dyskinesia (n = 8), decreased AEs (n = 6), on-quality (n = 3), psychological stability (n = 3) and sleeping well (n = 1).

### Secondary outcomes

Their mean mUPDRS, H&Y, ESS, sleep quality, PGI, compliance and AEs were not statistically different between the two regimens (Table 2).

**Table 2:** Secondary outcomes

	**Once-daily**	**Twice-daily**	***P *Value**
mUPDRS	17.5 ± 8.2	17.1 ± 8.9	0.324
Hoehn and Yahr stage	2.1 ± 0.6	2.1 ± 0.6	0.260
Excessive daytime sleepiness	6.3 ± 5.2	6.4 ± 4.8	0.498
Compliance (%)	98.4 ± 3.9	97.6 ± 3.8	0.112
Sleep questionnaire			
Overall quality of sleep	2.9 ± 2.6	3.2 ± 2.5	0.307
Nocturnal off-symptoms	2.9 ± 3.2	3.1 ± 3.2	0.396
Early morning off symptoms	2.5 ± 3.3	2.9 ± 3.2	0.384
PGI-I, no (%)			
Overall	32 (52.5)	42 (68.9)	0.078
Off duration	27 (44.3)	38 (62.3)	0.035^a^
Worst wearing-off severity	28 (45.9)	34 (55.7)	0.238
Dyskinesia duration	13 (21.3)	16 (26.2)	0.549
Dyskinesia severity	16 (26.2)	15 (24.6)	1.000

^a^*P* <0.005.

mUPDRS, Unified Parkinson’s Disease Rating Scale part 3; PGI-I, patient global impressions of improvement.

As for PGI, 50/61 reported improvement after switching to RPR from the IR form of agonists, whereas none reported deterioration in PGI for both regimens. Therefore, RPR was able to achieve significant improvement over the IR form of agonists. In the once-daily period, 8.2% (n = 5) had moderate improvement, 44.3% (n = 27) mild improvement, 32.8% (n = 20) no change, 13.1% (n = 8) mild deterioration, and 1.6% (n = 1) moderate deterioration. In the twice-daily period, 1.6% (n = 1) had marked improvement, 14.8% (n = 9) moderate improvement, 52.5% (n = 32) mild improvement, 18.0% (n = 11) no change, 9.8% (n = 6) mild deterioration, and 3.3% (n = 2) moderate deterioration. At the end of each period, the proportions of PGI-I were not statistically different (*P* = 0.078, Table 2).

For wearing off, in the once-daily regimen, 44.3% (n = 27) had decreased off-duration and 14.8% (n = 9) had increased off-duration. In the twice-daily regimen, the off-duration was decreased in 62.3% (n = 38) and increased in 6% (n = 9.8) of the subjects. The PGI-I of off-duration was significantly higher in twice-daily regimen (*P* = 0.035). For the worst wearing-off quality, there was no significant difference in PGI-I (*P* = 0.238). For dyskinesia, the duration and severity had no statistical difference in the once-daily and twice-daily regimens (*P* = 0.549 and *P* = 1.000).

### Adverse events

The incidence of drug-related AEs did not differ between the once-daily (54.1%, 33/61) and twice-daily (45.9%, 28/61) regimens (*P* = 0.227, Additional file 2). The most common drug-related AEs for the respective regimens were constipation (respectively, once-daily vs. twice-daily, 32.8%, 20/61 vs. 31.1%, 19/61), nausea (14.8%, 9/61 vs. 11.5%, 7/61), dyspepsia (11.5%, 7/61 vs. 9.8%, 6/61), dizziness (4.9%, 3/61 vs. 6.6%, 4/61), headache (4.9%, 3/61 vs. 1.6%, 1/61), aggravated REM sleep behavior disorder (3.3%, 2/61 vs. 3.3%, 2/61), hypersomnolence (1.6%, 1/61 vs. 3.3%, 2/61), dry mouth (3.3%, 2/61 vs. 4.9%, 3/61), agitation (1.6%, 1/61 vs. 1.6%, 1/61), and vivid dream (1.6%, 1/61 vs. 0%, 0/61).

Sixty patients chose to remain on RPR, but only one patient reverted back to pramipexole IR to take at the same time with other antiparkinsonian medications.

## Discussion

RPR is a once daily oral dopamine agonist for PD. In general, it is prescribed as a once daily regimen. When patients switched from once-daily to twice-daily or twice-daily to once-daily, however, their mean mUPDRS, H&Y, ESS, sleep quality and AEs were not statistically different. Despite the different dosing frequency, these findings indicate the same dose of RPR had a similar efficacy without an increase in AEs and was not inferior.

To check for bias of the sequence effect, we analyzed the outcomes based on the order. When the once-daily regimen was administered first (sequence 1 → 2), 51.79% (15/29) of the patients preferred the twice-daily regimen. The proportion of subjects who preferred the twice-daily regimen more was 50% (16/32) when the twice-daily regimen was administered first (sequence 2 → 1). Thus there was no sequence effect. We also did not find a sequence effect in the secondary outcome analysis.

For PGI-I, 50 patients reported improvement after RPR over the IR form of agonists. Admittedly, the dose was increased from 9.1 ± 5.0 mg of the IR form of agonists to 10.7 ± 6.2 mg of RPR. It is to be noted that we tried to optimize the medication with the available IR form of agonists balancing many factors such as motor fluctuation, dyskinesia and adverse effects. Therefore, it is definitely a benefit of RPR when the condition of patients’ improved even with the increased mean daily dose being within tolerance.

In our study, PD patients preferred the twice-daily regimen to the once-daily regimen (51% vs. 28%). In patients who preferred the twice-daily regimen, their main reasons were decreased severity of off symptoms or dyskinesia and decreased AEs. Especially for wearing-off duration, the PGI-I was significantly higher in twice-daily regimen. Thus, we should consider the twice daily regimen with its reduced severity of symptoms or decreased intolerable AEs for antiparkinsonian medications.

When patients chose the once-daily regimen, their main reason was improved on quality and decreased off symptoms or dyskinesia. Thus, we should consider the once daily regimen preferentially for young patients who are active workers in the daytime or have diphasic dyskinesia.

We expected changes in sleep quality and daytime sleepiness between the two regimens. However, there were no significant differences between the once-daily and twice-daily regimens.

As can be seen in the time window of the ropinirole level from Tompson et al. [5], the nocturnal concentration may be lower in the once daily RPR than the three times daily RIR. The down sloping of the ropinirole level was designed taking into consideration natural dopaminergic stimulation and improvement in nocturnal side effects including insomnia and hallucinations. Additionally, the reason was not fully explained by the time window of the ropinirole level in the steady state. However, multiple dosing of RPR may provide even better control in some patients.

Although patients showed no differences in their mean mUPDRS, H&Y, ESS, sleep quality and AEs, more patients preferred the twice-daily regimen and their main reason was more tolerable off-symptoms. Three of our study patients preferred the twice-daily regimen due to psychological stability, and decreased anxiety due to reduced off symptoms. This finding shows that psychological factors may explain the preference. Patients in this study previously maintained dopaminergic therapy with multiple dosing. They endured off symptoms and have fear of them. Therefore, as an ostensible reason of preference, they chose “more tolerable off-symptoms”, however the real reason would be “decreased anxiety due to reduced off symptoms.”

In antiparkinsonian medication, once daily dosing may improve compliance and achieve the desired outcome [5]. Four patients chose the once daily regimen due to convenience. However, almost all PD patients are taking multiple antiparkinsonian medications many times a day. Thus, compliance may not be a big issue especially for advanced PD patients.

## Conclusions

In this study, RPR was able to achieve improvement over the IR form of agonists. Twice-daily RPR was preferred over once-daily dosing in many patients.

This study has limitations as an open-label trials and pharmacodynamical study was not done. However, multiple dosing of RPR might be considered a therapeutic option if once-daily dosing is unsatisfactory. Greater satisfaction with twice-daily RPR may lead to achieving good compliance and improving outcomes.

In response to the final preference questionnaire, 28% (n = 17) of the patients preferred the once-daily regimen, 51% (n = 31) preferred the twice-daily regimen, and 21% (n = 21) did not have a preference.

## Abbreviations

(PD): Parkinson’s disease; (IR): Immediate-release; (PR): Prolonged-release; (RPR): Ropinirole prolonged release; (RPR): Ropinirole immediate-release; (mUPDRS): Unified Parkinson’s disease rating scale part 3; (H&Y): Hoehn and yahr stage; (ESS): Epworth sleep scale; (PGI): Patient global impression; (AEs): Adverse events; (PGI-I): Patient global impression of improvement.

## Competing interest

BSJ has received funding for travel from Norvartis Korea and GlaxoSmithKline Korea and has received research support as PI from Norvartis, Boehringer Ingelheim, Ipsen, the Korea Health 21 R&D project, Ministry of Health & Welfare, Republic of Korea (A101273), the National Research Foundation of Korea(NRF), Ministry of Education, Science and Technology (2010–0021653), ABRC (Advanced Biometric Research Center), KOSEF (Korean Science and Engineering Foundation), Seoul National University Hospital, the Mr. Chung Suk-Gyoo and Sinyang Cultural Foundation, and the Song Foundation. Other authors have no financial disclosures.

## Authors’ contributions

JYY, HJK and JBS designed the study, wrote the study protocol. JYY, JYL, YEK, JSK, JMK and JBS collected the data. JYY, JYL, YEK and JBS analyze and interpreted the data. JYY, HJK and JBS actively contributed to the writing. All authors reviewed the manuscript and approved the final version.

## Pre-publication history

The pre-publication history for this paper can be accessed here:

http://www.biomedcentral.com/1471-2377/13/113/prepub

## Supplementary Material

Additional file 1 Dose switches from conventional dopamine agonists to ropinirole PR. Click here for file

Additional file 2 Adverse events. Click here for file

## References

[B1] Obeso JA Rodriguez-Oroz MC Chana P Lera G Rodriguez M Olanow CW The evolution and origin of motor complications in Parkinson’s disease Neurology 2000 55 11 Suppl 4 S13 S20 discussion S21-13 11147505

[B2] Stocchi F Hersh BP Scott BL Nausieda PA Giorgi L Ease PDMSI Ropinirole 24-hour prolonged release and ropinirole immediate release in early Parkinson’s disease: a randomized, double-blind, non-inferiority crossover study Curr Med Res Opin 2008 24 10 2883 2895 10.1185/03007990802387130 18768106

[B3] Stocchi F Giorgi L Hunter B Schapira AH PREPARED: Comparison of prolonged and immediate release ropinirole in advanced Parkinson’s disease Mov Disord 2011 26 7 1259 1265 10.1002/mds.23498 21469195

[B4] Grosset D Antonini A Canesi M Pezzoli G Lees A Shaw K Cubo E Martinez-Martin P Rascol O Negre-Pages L Adherence to antiparkinson medication in a multicenter European study Mov Disord 2009 24 6 826 832 10.1002/mds.22112 19191340

[B5] Tompson DJ Vearer D Steady-state pharmacokinetic properties of a 24-hour prolonged-release formulation of ropinirole: results of two randomized studies in patients with Parkinson’s disease Clin Ther 2007 29 12 2654 2666 10.1016/j.clinthera.2007.12.010 18201581

[B6] Jost WH Buhmann C Fuchs G Greulich W Hummel S Korchounov A Mungersdorf M Schwarz M Spiegel-Meixensberger M Initial experience with ropinirole PR (prolonged release) J Neurol 2008 255 Suppl 5 60 63 18787884 10.1007/s00415-008-5008-z

[B7] Onofrj M Bonanni L De Angelis MV Anzellotti F Ciccocioppo F Thomas A Long half-life and prolonged-release dopamine receptor agonists: a review of ropinirole prolonged-release studies Parkinsonism Relat Disord 2009 15 Suppl 4 S85 S92 20123565 10.1016/S1353-8020(09)70842-9

