# Supplementary material for: Comparison of once-daily versus twice-daily combination of Ropinirole prolonged release in Parkinson’s disease
Source: BMC Neurol. 2013 Sep 2;13:113. doi: 10.1186/1471-2377-13-113 (PMC3766261; doi:10.1186/1471-2377-13-113)
Supplement: Additional file 1 — Dose switches from conventional dopamine agonists to ropinirole PR. [file 1471-2377-13-113-S1.docx]

**Additional file 1.** Dose switches from conventional dopamine agonists to ropinirole PR.

| Ropinirole IR → Ropinirole PR | | | Pramipexole IR → Ropinirole PR | | |
| --- | --- | --- | --- | --- | --- |
| Ropinirole IR daily dose, mg | Ropinirole PR dose, mg | | Pramipexole IR daily dose, mg | Ropinirole PR dose, mg | |
|  | Once daily | Twice daily |  | Once daily | Twice daily |
| 0.75 | 2.0 |  | 0.1875 | 2.0 |  |
| 1.5 | 2.0 |  | 0.375 | 2.0 |  |
| 2.0 | 4.0 | 2.0 – 2.0 | 0.5625 | 4.0 | 2.0 – 2.0 |
| 3.0 | 4.0 | 2.0 – 2.0 | 0.75 | 4.0 | 2.0 – 2.0 |
| 4.0 | 4.0 | 2.0 – 2.0 | 1.0 | 6.0 | 4.0 – 2.0 or 2.0 – 4.0 |
| 6.0 | 6.0 | 4.0 – 2.0 or 2.0 – 4.0 | 1.125 | 6.0 | 4.0 – 2.0 or 2.0 – 4.0 |
| 7.5 | 8.0 | 4.0 – 4.0 | 1.5 | 8.0 | 4.0 – 4.0 |
| 8.0 | 8.0 | 4.0 – 4.0 | 2.0 | 12.0 | 6.0 – 6.0 |
| 9.0 | 10.0 | 6.0 – 4.0 or 4.0 – 6.0 | 2.25 | 12.0 | 6.0 – 6.0 |
| 12.0 | 12.0 | 6.0 – 6.0 | 2.5 | 16.0 | 8.0 – 8.0 |
| 15.0 | 16.0 | 8.0 – 8.0 | 3.0 | 16.0 | 8.0 – 8.0 |
| 18.0 | 16.0 | 8.0 – 8.0 | 4.0 | 24.0 | 12.0 – 12.0 |
| 21.0 | 20.0 | 10.0 – 10.0 | 4.5 | 24.0 | 12.0 – 12.0 |
| 24.0 | 24.0 | 12.0 – 12.0 |  |  |  |

IR, immediate-release; PR, prolonged-release.
