# Supplementary material for: Comparison of once-daily versus twice-daily combination of Ropinirole prolonged release in Parkinson’s disease
Source: BMC Neurol. 2013 Sep 2;13:113. doi: 10.1186/1471-2377-13-113 (PMC3766261; doi:10.1186/1471-2377-13-113)
Supplement: Additional file 2 — Adverse events. [file 1471-2377-13-113-S2.docx]

**Additional file 2.** Adverse events

|  | Baseline | Once-daily | Twice-daily |
| --- | --- | --- | --- |
| Adverse events (%) | 25 (41.0) | 32 (52.5) | 28 (45.9) |
| Nausea | 5 ( 8.2) | 9 (14.8) | 7 (11.5) |
| Dizziness | 6 ( 9.8) | 3 ( 4.9) | 4 ( 6.6) |
| Somnolence | 1 ( 1.6) | 1 ( 1.6) | 2 ( 3.3) |
| Headache | 0 ( 0.0) | 3 ( 4.9) | 1 ( 1.6) |
| Constipation | 18 (29.5) | 20 (32.8) | 19 (31.1) |
| Dyspepsia | 3 ( 4.9) | 7 (11.5) | 6 ( 9.8) |
| Fatigue | 0 ( 0.0) | 0 ( 0.0) | 0 ( 0.0) |
| Hallucination | 0 ( 0.0) | 0 ( 0.0) | 0 ( 0.0) |
| Others | 3 ( 4.9) | 6 ( 9.8) | 6 ( 9.8) |
